# Digital health tools for the passive monitoring of depression: a systematic review of methods

**DOI:** 10.1038/s41746-021-00548-8

**Published:** 2022-01-11

**Authors:** Valeria De Angel, Serena Lewis, Katie White, Carolin Oetzmann, Daniel Leightley, Emanuela Oprea, Grace Lavelle, Faith Matcham, Alice Pace, David C. Mohr, Richard Dobson, Matthew Hotopf

**Affiliations:** 1 Institute of Psychiatry, Psychology and Neuroscience, King’s College London, London, UK; 2 NIHR Maudsley Biomedical Research Centre, South London and Maudsley NHS Foundation Trust, London, UK; 3 Department of Psychology, University of Bath, Bath, UK; 4 Chelsea And Westminster Hospital NHS Foundation Trust, London, UK; 5 Center for Behavioral Intervention Technologies, Northwestern University, Feinberg School of Medicine, Chicago, IL USA; 6 Department of Preventive Medicine, Northwestern University, Feinberg School of Medicine, Chicago, IL USA; 7 Department of Biostatistics and Health Informatics, Institute of Psychiatry, Psychology and Neuroscience (IoPPN), King’s College London, 16 De Crespigny Park, London, SE5 8AF UK

**Keywords:** Human behaviour, Diagnostic markers, Machine learning

## Abstract

The use of digital tools to measure physiological and behavioural variables of potential relevance to mental health is a growing field sitting at the intersection between computer science, engineering, and clinical science. We summarised the literature on remote measuring technologies, mapping methodological challenges and threats to reproducibility, and identified leading digital signals for depression. Medical and computer science databases were searched between January 2007 and November 2019. Published studies linking depression and objective behavioural data obtained from smartphone and wearable device sensors in adults with unipolar depression and healthy subjects were included. A descriptive approach was taken to synthesise study methodologies. We included 51 studies and found threats to reproducibility and transparency arising from failure to provide comprehensive descriptions of recruitment strategies, sample information, feature construction and the determination and handling of missing data. The literature is characterised by small sample sizes, short follow-up duration and great variability in the quality of reporting, limiting the interpretability of pooled results. Bivariate analyses show consistency in statistically significant associations between depression and digital features from sleep, physical activity, location, and phone use data. Machine learning models found the predictive value of aggregated features. Given the pitfalls in the combined literature, these results should be taken purely as a starting point for hypothesis generation. Since this research is ultimately aimed at informing clinical practice, we recommend improvements in reporting standards including consideration of generalisability and reproducibility, such as wider diversity of samples, thorough reporting methodology and the reporting of potential bias in studies with numerous features.

## Introduction

Depression remains the leading cause of disability worldwide^1^, with a largely chronic course and poor prognosis^2^. Early recognition and access to treatment, as well as a better trial methodology, have been linked to improved treatment outcomes and prognosis^3^.

The use of digital technology to track mood and behaviour brings enormous potential for clinical management and the improvement of research in depression. By passively sensing motion, heart rate and other physiological variables, smartphone and wearable sensors provide continuous data on behaviours that are central to psychiatric assessment, such as sociability^4^, sleep/wake cycles^5^, cognition, activity^6^ and movement^7^.

With the global trend toward increased smartphone ownership (44.9% worldwide, 83.3% in the UK) and wearable device usage forecast to reach one billion by 2022^8^, this new science of “remote sensing”, sometimes referred to as digital phenotyping or personal sensing^9^ presents a realistic avenue for the management and treatment of depression. When combined with the completion of questionnaires, remote sensing may generate more objective and frequent measures of mood and other core dimensions of mental disorders, instead of relying on retrospective accounts of patients or participants.

The first step in generating meaningful clinical information from data derived from digital sensors is to generate features, which are the smallest constructed building blocks, designed to explain the behaviours of interest (see Mohr et al. ^10^ for a detailed analytical framework). These low-level features are often aggregated to define high-level behavioural markers, which can be understood as symptoms. For example, GPS data (sensor), can be translated into ‘location type’ (low-level feature), ‘increased time at home location’ (high-level behaviour) derived from location data may indicate social withdrawal or lack of energy (symptom), and may therefore be associated with depression severity.

One of the main challenges that arise from this emerging field is that it sits at the intersection between computer science, engineering, and clinical science. The advantages of a multidisciplinary approach are evident, but these domains are yet to be brought together efficiently^11,12^, giving rise to large differences in reporting standards with the risk that reproducibility may be threatened^13^.

Previous reviews in affective disorders cite the level of heterogeneity across studies as a barrier to carrying out meta-analytic syntheses of the results. Additionally, these reviews have included non-validated measures of depression, and a mix of bipolar and unipolar samples, characteristics which not only show divergent results^11,12,14^, but add study diversity. There is therefore a need for a comprehensive review of methodologies, with more specific inclusion criteria, to highlight the sources of heterogeneity and methodological shortcomings in the field.

Given the difficulty in extracting a clear message from the available literature, the current work aims to review studies linking passive data from smartphone and wearable devices with depression and summarise key methodological aspects, to: (a) identify sources of heterogeneity and threats to reproducibility, and (b) identify leading digital signals for depression. We will also assess the quality of the included studies and evaluate their reporting of the feasibility of passive data collection methods, participant retention and missing data.

## Results

**Fig. 1:**
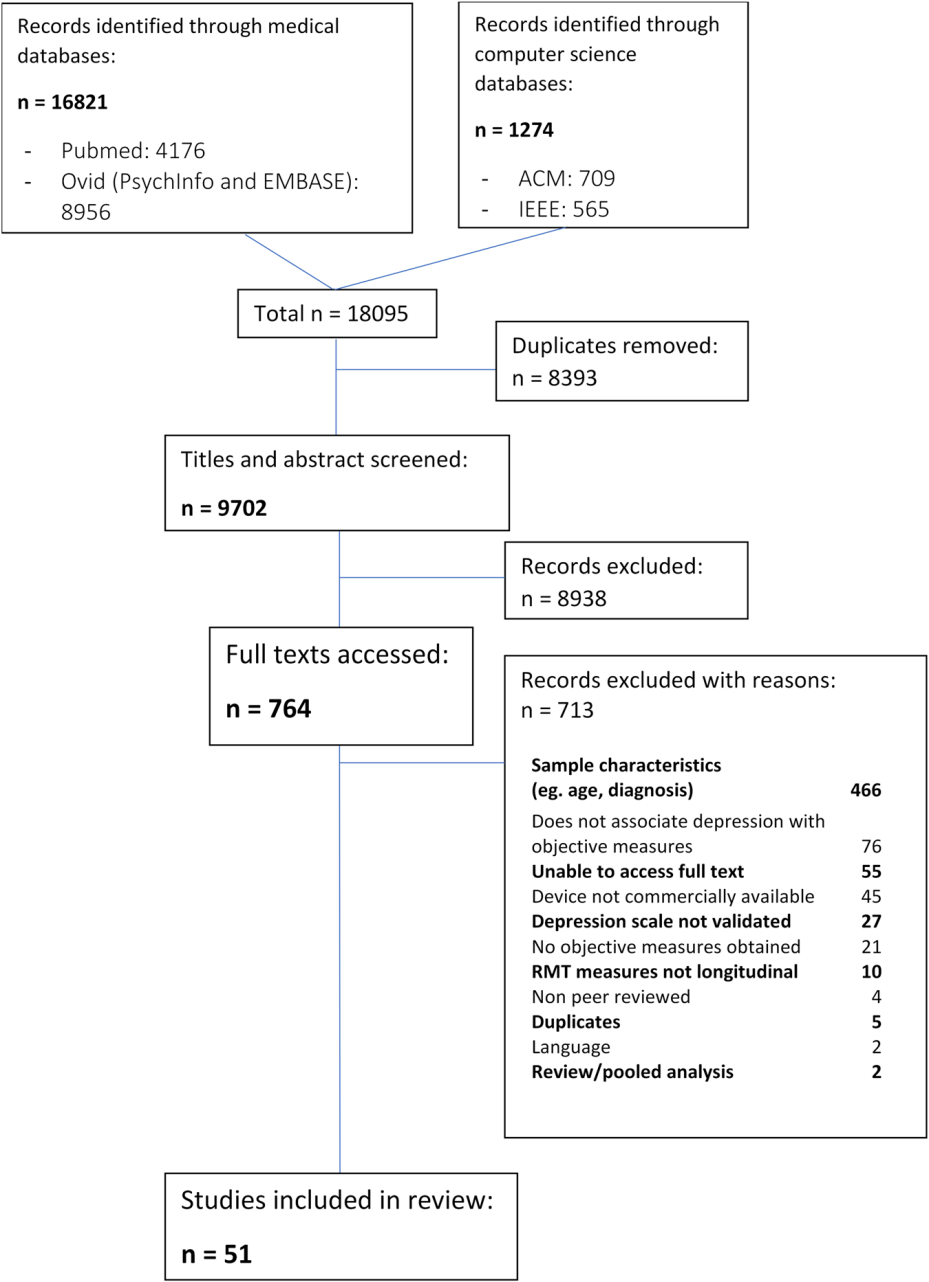
Study selection flowchart. Medical and computer science databases were searched to ensure relevant fields were covered. The current flowchart lists reasons for excluding the study from the data extraction and quality assessment.

Fifty-one studies were included in the review (see Fig. 1). The majority of articles (*n* = 45) were published in medical journals, and 33 (65%) were from North America. A summary of included studies is presented in Table 1.

**Table 1:** Summary characteristics of included studies.

First author	Year	Country	Field	*N* (RMT)^a^	% female	Mean age (range/SD)	RMT^a^ follow up (days)	Sample type	Depression measure	Passive feature type								
										Sleep	Physical activity	Circadian rhythm	Sociability	Location	Phone use	Physiological	Environmental	Total feature types
Avila-Moraes^36^	2013	Brazil	M	30	100.0	44 (18–60)	7	Clinical	BDI, HAMD, MADRS		x	x				x		3
Ben-Zeev^46^	2015	USA	M	37	21.0	22.5 (19–0)	70	Student	PHQ-9	x	x		x	x				4
Boukhechba^34^	2018	USA	M	72	51.4	19.8 (2.4)	14	Student	DASS-21		x		x	x				3
Burns^19^	2011	USA	M	7	87.5	37.4 (19–51)	56	Community	PHQ-9	x	x		x	x	x			5
Byrne^25^	2019	Australia	M	42	0.0	(18–29)	7	Community	SCRAM - dep	x	x	x						3
Caldwell^76^	2019	USA	M	115	100.0	27.5 (6.1)	3	Community	BDI-II	x								1
Cho^4^	2016	South Korea	M	532	56.0	57	720	Community	BDI-II				x					1
David^47^	2018	USA	M	132	60.0	20.68 (18–21)	7	Student	PHQ-4				x		x			2
Difrancesco^20^	2019	Netherlands	M	359	62.4	50.1 (11.1)	7	Community	BDI-II	x	x	x						3
Dillon^21^	2018	Ireland	M	396	50.8	nr	7	Clinical	CES-D		x							1
Doane^77^	2015	USA	M	76	76.0	18.1 (0.4)	3	Student	CES-D	x								1
Doryab^44^	2014	USA	M	6	33.3	nr	120	Student	CES-D				x	x			x	3
Ghandeharioun^6^	2017	USA	CS	12	75.0	37 (20–73)	56	Clinical	HAM-D	x	x		x	x	x	x		6
Haeffel^78^	2017	USA	M	47	55.3	20.9	7	Student	BDI-II	x								1
Hori^79^	2016	Japan	M	40	52.5	39.8	7	Clinical	HAM-D	x								1
Jacobson^80^	2019	Brazil	M	15	87.0	47.6 (10.5)	7	Clinical	BDI, HAMD	x	x							2
Kawada^32^	2007	Japan	M	105	29.5	24.1 (1.8)	4	Student	CES-D	x	x	x						3
Knight^81^	2018	Australia	M	23	77.0	20.7 (3.2)	3	Community	DASS-21		x							1
Li^82^	2018	Australia	M	375	53.9	59.5 (5.5)	7	Community	CES-D		x							1
Lu^5^	2018	USA	CS	103	76.7	(18–25)	nr	Student	QIDS	x	x			x				3
Luik^83^	2013	Netherlands	M	1734	53.4	62.3 (9.4)	7	Community	CES-D	x								1
Luik^30^	2015	Netherlands	M	1714	53.6	62.2 (9.4)	7	Community	CES-D	x								1
McCall^84^	2015	USA	M	58	67.0	42.1 (12.4)	56	Clinical	HAM-D	x								1
Mendoza-Vasconez^85^	2019	USA	M	266	nr	40.6 (9.9)	7	Community	HAM-D		x							1
Moukaddam^27^	2019	USA	M	22	76.0	50.3 (10.1)	56	Clinical	PHQ-9		x		x					2
Naismith^86^	2011	Australia	M	44	43	62.3	14	Clinical	HAM-D	x								1
Park^87^	2007	USA	M	54	57.4	43 (21–76)	14	Community	CES-D	x	x							2
Pillai^88^	2014	USA	M	39	73.8	55 (3.2)	7	Student	BDI-II	x								1
Pratap^89^	2019	USA	M	271	77.8	33.4 (10.7)	90	Community	PHQ-2				x	x				2
Robillard^33^	2013	Australia	M	66	62.7	21.5	7	Clinical	clinician assessment	x								1
Robillard^41^	2014	Australia	M	238	64.3	40.4	10	Clinical	HAM-D	x		x						2
Robillard^38^	2015	Australia	M	342	55.1	22.3	14	Clinical	clinician assessment	x		x						2
Robillard^90^	2016	Australia	M	25	48.0	20.9 (4.6)	14	Clinical	clinician assessment	x		x						2
Robillard^91^	2018	USA	M	12	58.0	20.1 (18–31)	13	Clinical	clinician assessment	x								1
Saeb^7^	2015	USA	M	21	71.4	28.9 (19– 58)	14	Student	PHQ-9			x		x	x			3
Saeb^42^	2016	USA	M	38	20.8	nr	70	Community	PHQ-9		x			x				2
Sano^22^	2018	USA	M	47	72.0	(18– 25)	30	Student	MCSF-12	x	x	x	x	x	x	x		7
Slyepchenko^37^	2019	Canada	M	70	57.9	(18– 65)	15	Clinical	MINI	x	x	x						3
Smagula (a)^39^	2018a	USA	M	145	67.0	60 (36-82)	9	Community	HAM-D			x						1
Smagula (b)^92^	2018	USA	M	45	38.8	38.08	10	Community	HAM-D			x						1
Stremler^93^	2017	Canada	M	101	62.7	34.1	5	Community	CES-D	x								1
Tao^35^	2019	China	M	220	52.3	20.3 (2.4)	7	Student	PROMIS - dep		x							1
Vallance^94^	2013	Canada	M	385	0.0	65.3 (7.5)	3	Community	CES-D		x							1
Vanderlind^95^	2014	USA	M	35	42.3	19.8 (18–23)	21	Student	CES-D	x		x						2
Wahle^23^	2016	Switzerland	M	36	64.3	(20–57)	14	Community	PHQ-9		x		x	x	x			4
Wang^26^	2014	USA	CS	48	20.8	nr	7	Student	PHQ-9	x			x	x				3
Wang^96^	2018	USA	CS	83	51.8	20.1 (2.3)	126	Student	PHQ-8	x	x		x	x	x	x		6
White^40^	2017	USA	M	418	60.3	57 (35–85)	7	Community	CES-D	x		x						2
Yang^45^	2017	China	CS	48	nr	nr	70	Student	PHQ-9				x					1
Yaugher^97^	2015	USA	M	100	58.3	18.6 (18– 27)	7	Student	PAI-dep	x								1
Yue^18^	2018	USA	CS	54	nr	(18–25)	nr	Student	PHQ-9		x			x				2
*N* = 52				**Median**	**Median**	**Median**	**Median**		**Total** ***N***	**Total**								
				58.0	57.9	37.2	9		16	31	24	14	14	14	7	4	1	

*RMT* remote measurement technologies, *SD* standard deviation, *M* medical field, *CS* computer science field, *BDI* Beck’s Depression Inventory, *HAM-D* Hamilton Depression Rating Scale, *MADRS* Montgomery–Åsberg Depression Rating Scale, *PHQ* Patient Health Questionnaire, *PAI-dep* Personality Assessment Inventory-depression subscale, *CES-D* Center for Epidemiologic Studies Depression Scale, *MINI* Mini International Neuropsychiatric Interview, *PROMIS* Patient-Reported Outcomes Measurement Information System, *MCSF-12* Mental Component of the Short Form Health Survey, *QIDS* Quick Inventory of Depressive Symptomatology, *DASS* Depression Anxiety Stress Scales, *SCRAM* sleep, circadian rhythms, and mood questionnaire.

^a^Number of participants/length of follow-up included in passive data collection samples; these may be lower than overall study sample sizes.

Studies were evenly divided between community samples (*n* = 19), student samples (*n* = 18) and clinical populations (*n* = 14). The median sample size was 58, the median age of participants was 38 years, and the median percentage of females was 58%. However, there was a striking lack of information on some key data—with 12% and 8% of studies failing to give data on age or gender, respectively, and 63% failing to include information on ethnicity. Computer science journals were less likely to report age and gender but more likely to report ethnicity (33% studies failing to report each demographic). Fifteen different measures of depression were used, the most commonly used scales being the Center for Epidemiological Studies Depression Scale (CES-D^15^; *n* = 12 studies), Hamilton Rating Scale for Depression (HAM-D^16^; *n* = 12 studies), and Patient Health Questionnaire-9 (PHQ-9^17^; *n* = 9). There were 14 types of devices used across all studies: 12 of them actigraphy-based wrist-worn devices including one Fitbit and a Microsoft band, as well as one pedometer and smartphones (both android and iPhone). For a breakdown of devices, models and sensors used to measure behaviour see Supplementary Table 1.

**Fig. 2:**
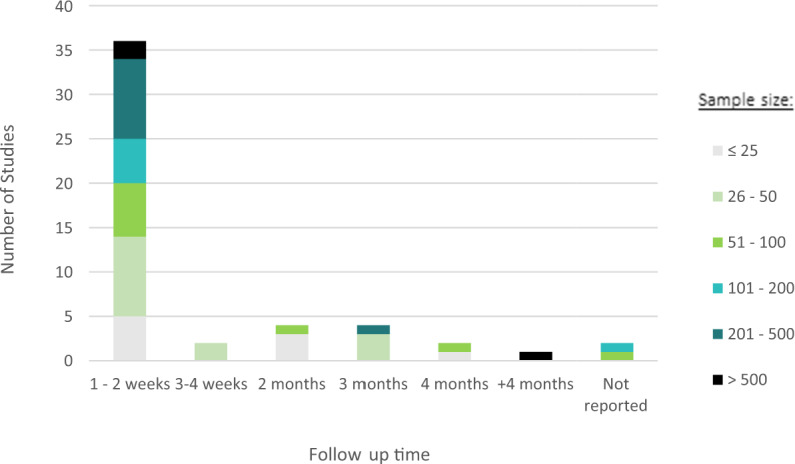
Sample sizes and follow-up times for all included studies. The number of studies by the length of time participants were followed up for in each study, differentiated by sample size.

Most studies had a cohort design, meaning that depression was measured at least at two different time points (see Table 2). However, these time points tended to be shorter than 2 weeks (Fig. 2). Two studies provided no information on the length of follow-up, instead only mentioning that data was obtained from participants providing at least 72 h of consecutive data^5,18^.

**Table 2:** The breakdown of study designs within each sample type.

Study design	Total	Student	Community	Clinical
Cross-sectional	19	4	10	5
Case-control	6	0	1	5
Cohort	25	14	6	3
RCT	3	0	2	1
Total	51	18	19	14

To understand the relationships between depression and objective features, studies either looked at group differences (including classification analyses) or correlation and regression. Most studies presented direct bivariate relationships (*n* = 45), allowing for a closer evaluation of which features are promising markers of depressive symptomatology. Ten studies presented the result of a combination of features and their association with the depressive state (*n* = 7), or depression severity (*n* = 8), using machine learning methods. Bivariate Pearson correlation coefficients were the most used analytical method (*n* = 32).

### Quality assessment and feasibility

**Fig. 3:**
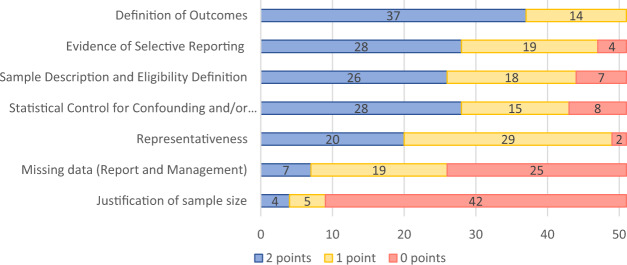
Quality of the literature by each domain. The figure shows the number of studies scoring on each study quality item. 2 points are given for fully addressing quality criteria, 1 point for partially addressing quality criteria, and 0 points for failing to address quality criteria.

Figure 3 shows a breakdown of quality scores for each item (see Supplementary Fig. 1 and Supplementary Table 2 for quality assessment scores per study). Justification of sample size was rarely given, and sample representativeness was poor, possibly reflecting that many reports were pilot or feasibility studies. Recruitment strategies and non-participation rates were not reported in the majority of cases. Missing data and strategies for handling missing data were infrequently described. Only four studies referred to a previously published protocol^19–22^.

Only five studies reported engagement rates at follow-up, and they all measured engagement at different time points, making comparisons difficult. Additionally, sensor data was sometimes obtained for a subsample, whereas acceptability measures were reported for the wider sample. Eighteen studies (35%) reported, or provided enough information to calculate, how many participants completed the study—results ranging from 22% adherence to the study^23^ at 4 weeks, to 100%^24^, with a median of 86.6% completers.

Reasons for dropouts were provided in four studies and were due to equipment malfunction and technical problems using devices^19,25–27^ . Six additional studies reported issues including; lack of data for consecutive days, software error, participants forgetting to charge phones or devices, server and network connectivity problems, sensors breaking, missing clinical data which impeded comparisons with sensor data, and mobile software updates, which can interfere with data integrity^7,22,28–31^.

### Associations between objective features and depression

**Fig. 4:**
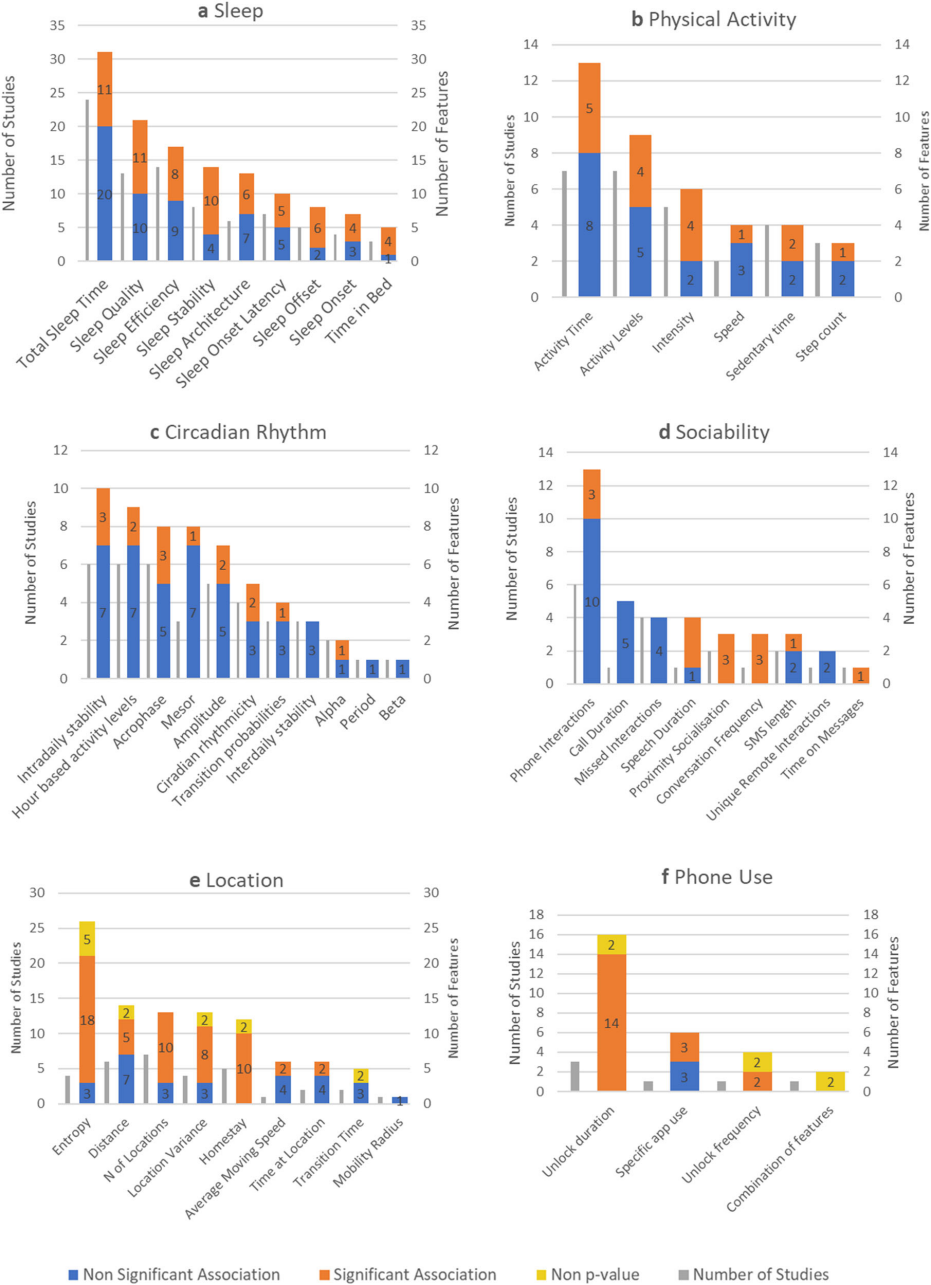
Feature associations with depression by behaviour type. The number of times each feature (**a** sleep, **b** physical activity, **c** circadian rhythm, **d** sociability, **e** location and **f** phone use) has been reported in all included studies and their association with depression, where these associations are defined as having a below-threshold *p*-value (“Significant Association”), above-threshold *p*-value (“Non-Significant Association”), and where statistical methods have been used that do not yield *p*-values (“Non-*p*-value”). The graphs also show the number of studies assessing each feature.

The association between groups of features and depression is given in Fig. 4, broken down by feature type. We give the number of studies that have reported the feature and the number of feature–depression associations that reached statistical significance as a proportion of the total such associations reported. See Supplementary Tables 3–10 for a list of tables with terms and feature definitions.

Twenty-nine studies collected data on sleep, typically ascertained using accelerometer, light and heart rate sensors. Nine different features of sleep are reported in Fig. 4A. Sleep quality, encompassing features relating to sleep fragmentation (number of awakenings and wake after sleep onset [WASO]), was the most commonly reported feature. Sleep efficiency is presented as a separate feature given its prevalence in studies. For all significant results, lower sleep efficiency or quality was associated with higher depression scores. Features with higher proportions of significant findings are features of sleep stability, sleep offset, time in bed; longer time in bed and later sleep offset were associated with higher depression scores.

Across studies finding significant results, sleep variability was higher for those with depression compared to controls (27), and those with more severe symptoms (28). The average length of follow-up for studies showing significant associations between sleep stability and depression was 24.7 days (range = 4–63), whereas that for studies showing no significant associations was 8.6 (range = 3–21).

Total sleep time showed mixed directionality of significance, with some studies finding negative correlations between total sleep time and higher depression^26,32^, others finding the depressed group having longer sleep time than controls^33^.

Measures of physical activity were collected in 19 studies using a mixture of smartphone (*n* = 8) and wearable devices (*n* = 11). Activity levels were predominantly measured as a gross motor activity within a day, and showed that depression was negatively correlated with physical activity^20,34^. Out of the seven studies extracting ‘activity levels’ as a feature within physical activity, both studies using smartphones found a significant difference in depression severity, compared to one out of the five that used wrist actigraphy. Higher depressive symptoms were associated with less time spent engaging in physical activity^5^, movement speed^18^ and step count^27^. Two out of the three studies looking at intensity found lower depression in those with more instances of intense activity and fewer sedentary behaviours^5,20^, with the third study^35^ finding no significant associations. The authors reported very little variability in activity intensity, which could account for such findings.

A total of 13 studies assessed movement patterns within a 24-h period. All used accelerometery data, except for Saeb^7^ who used GPS data for circadian movement. All significant associations indicated that disturbed rest-activity patterns were associated with depressive symptoms, however, in the majority of instances where circadian rhythm was reported, no significant association with mood was detected. Depression has been associated with lower daytime activity and higher night-time activity (hour-based activity levels^36,37^), low intra-daily stability, more fragmented intra-daily movement, e.g., leaving for work and coming back at less regular times^7^, later acrophase, or later activity peaks^38–40^; lower amplitude, less difference between the average levels of activity during the peaks vs. the troughs of activity^20,39^. Four studies calculated circadian rhythmicity as a measure of the extent to which a participant’s pattern follows an expected Cosinor model, finding lower circadian rhythmicity more likely to be associated with being depressed^37,41–43^.

Eleven studies assessed sociability. The average number of ingoing and outgoing calls was found to be negatively correlated with depressive symptoms in one small study (*n* = 6), and only in men^44^. Yang et al.^45^, with a combination of microphone, GPS and Bluetooth sensing as a proxy for social proximity, found that an interaction between environmental noise and proximity to others was informative of depressive state, e.g. being in a quiet place with few people around, compared to either spending time outside alone or in a noisy environment with more than 3 people. Other studies found that a higher frequency of conversations in the day and at night correlated with lower depression^26^, as well as being around human speech for longer^46^.

Location was assessed in 11 studies, measured via GPS. In addition to traditional statistical analyses, Saeb et al.^7^ estimated accuracy and mean normalised residual mean square difference (NRMSD) to assess the performance of prediction models. We, therefore, do not have levels of significance as expressed via *p*-values for all features. Entropy was reported in 26 cases in four different studies. High entropy, or spending more time in fewer, more consistent locations, was associated with depression, as compared to lower entropy, where people spend more time in a greater number of more varied locations. Features of location variance—how varied a participant’s locations are—show a negative correlation with depression, where the more varied the locations, the lower the likelihood of being depressed. Homestay—the amount of time spent at home—shows one of the most consistent patterns across the field, with all included studies reporting a significant association with depression.

Three studies associated individual phone use features with depression. All studies found that increased unlock duration and unlock frequency were associated with depression, non-*p*-value tests reported a mean NRMSD of 0.268 and 0.249, and 74.2% and 68.6% accuracy in classifying depressed vs. non-depressed participants, respectively. Increased use of specific apps, such as Instagram, iOS maps, and the use of photo and video apps was associated with greater depression, whereas book apps were associated with milder symptoms^47^.

The temperature was measured by Ávila-Moraes et al.^36^, who extracted more than 5 skin temperature features from a wrist-worn device, and found depressed people to have a longer time of elevated temperature compared to controls. One study^48^ reported no association between heart rate and depression scores.

Ávila-Moraes et al.^36^ also used a wrist-worn actigraphy device to measure light exposure and extracted four features. She found depressed groups to have a lower variance of light intensity than controls. Another study found humidity to have a significant positive correlation with depressed symptoms *(r* = 0.4) in women, but a negative correlation in men, suggesting females, but not males might feel worsening in their condition during rainy weeks^44^.

### Sensitivity analysis

We carried out a sensitivity analysis to evaluate whether including only high-quality studies had any effect on our overall findings, the results of which can be found in Supplementary Fig. 3. After excluding studies with a score of eight out of 15 or lower, 20 papers remained. Overall, we found that excluding poor-quality studies did not change the patterns of association or significance ratios for sleep, physical activity, sociability, and location, beyond reducing the number of studies and therefore features that were analysed. Many of the studies on circadian rhythms are excluded, making existing associations even more tenuous; all studies showing a significant association between mood and intradaily stability or acrophase are lost, as are those finding no association between hour-based activity levels and depression. No studies looking at bivariate associations between phone use and depression remained.

### Combined features

Tables 3 and 4 show the ten studies combining digital features to predict symptom severity (regression models) or depressive state (classification models). Twenty-four models in total were presented by all studies, the majority of which (*n* = 18) included features of physical activity, followed by location (*n* = 14), phone use (*n* = 11) and sleep (*n* = 9). Both classification and regression models showed predictive value, however, many of them lacked information regarding the handling of missing sensor data and calibration. Those that do, report simple imputation methods such as mean imputation, with two studies using multiple imputation methods^6,18^.

**Table 3:** Details for studies analysing combined features using classification models.

Study ID	Quality rating	First Author, Year	Device	Groups	*N*	No. of features	Feature type	Algorithm/model	Performance measure	Discrimination value	Missing data handling	Validation method	Comparison models
1	12	Sano, 2018	Q sensor, smartphone	MCS SF-12 Low vs. High	47	204	PA, SC, Li	SVM RBF	Accuracy	85.1	Interpolation	10-fold cross-validation	LASSO, SVM Linear
						441	PA, L, PU, SC, ST	SVM RBF	Accuracy	86.1			
						700	S, PA, PU, SC, ST, HR, Cl	SVM RBF	Accuracy	77.2			
						296	S, PA, PU	SVM RBF	Accuracy	78.7			
						25	PU	SVM RBF	Accuracy	71.1			
						25	S	SVM RBF	Accuracy	65			
2	8	Yue, 2018	Android	Clinician MDD vs. HC	25	8	PA, L	SVM RBF	F1	0.66	Multiple Imputation	LOOCV	l2-regularised (ridge) regression
			iPhone		54	8	PA, L	SVM RBF	F1	0.76			
3	8	Wahle, 2016	Smartphone	PHQ-9 Dep vs. HC	36	120	PA, So, L, PU	Random Forest	Accuracy	60.1	Unclear	LOOCV	SVM
4	10	Pratap, 2019	Smartphone	PHQ-2 Dep vs. HC	93	10	So, L	Random Forest	Median AUC	>0.50 (for 80.6% sample)	Mean imputation	None	
5	7	Saeb, 2015	Android	PHQ-9 Dep vs. HC	18	8	CR, L	Elastic Net Logistic Regression	Accuracy	78.8	Unclear	LOOCV	
6	7	Wang, 2018	Smartphone	PHQ 4 Dep vs. HC	83	9	S, PA, L, PU, HR	Lasso Logistic Regression	AUC	0.809	Unclear	10-fold cross validation	
7	9	Lu, 2018	smartphone and Fitbit	QIDS	69	36	S, PA, So	Multi-Task Deep Learning	F1	0.77	Exclusion	LO(W)OCV	STL (Lasso) STL (Ridge), MTL Lasso and Ridge

*MCS SF* mental component survey short form, *PHQ* Patient Health Questionnaire, *MDD* major depressive disorder, *HC* healthy control, *S* sleep, *PA* physical activity, *CR* circadian rhythm, *So* Sociability, *L* Location, *PU* phone use, *SC* skin conductance, *ST* skin temperature, *HR* heart rate, *Li* light, *Cl* clinical data, *SVM RBF* Support Vector Machine - Radial Basis Function, AUC Area Under the Curve, LOOCV Leave One Out Cross Validation, STL Single Task Learning, MTL = Multi-Task Learning

**Table 4:** Details for studies analysing combined features using regression models.

Study ID	Quality rating	First Author, Year	Device	Outcome	*N*	No. of features	Feature type	Algorithm	Performance measure	Exact statistic	Missing data handling	Validation method	Comparison
2	8	Yue, 2018	Android	PHQ9	25	8	PA, L	SVM RBF	r	0.46	Multiple Imputation	LOOCV	Support Vector Multivariate Linear Regression
			iPhone	PHQ9	54	8	PA, L	SVM RBF	r	0.41			Support Vector Multivariate Linear Regression
4	10	Pratap, 2019	Smartphone	PHQ2	93	10	PA, So, L	Random Forests	R2	≈ 0	Mean Imputation	None Reported	
5	7	Saeb, 2015	Smartphone	PHQ9	18	8	CR, L	Elastic net Linear Regression	Mean NRMSD	0.251	Unclear	LOOCV	
					21	2	PU	Elastic net linear regression	Mean NRMSD	0.273			
6	7	Wang, 2018	Smartphone	pre PHQ 8	83	10	S, PA, L, PU, HR	Lasso Linear Regression	MAE	2.4	Unclear	10-fold cross validation	
				post PHQ 8	83	5	S, PA, So, L, PU	Lasso Linear Regression	MAE	3.6			
7	9	Lu, 2018	Smartphone, Fitbit	QIDS	69	36	S, PA, So	Multi-Task deep Learning	R2	0.44	Exclusion	LO(W)OCV	STL (Lasso) STL (Ridge), MTL Lasso and Ridge
8	7	Burns, 2011	Smartphone	PHQ9	7	38	PA, So, L, PU, Li	Regression Trees	Accuracy	nr	Unclear	10-fold cross validation	
9	8	Jacobson, 2019	Actiwatch	BDI-II	15	nr	PA, Li	Xgboost	r	0.86	Unclear	LOOCV	
10	7	Ghandeharioun, 2017	Empatica, Smartphone	HRDS	12	700	S, PA, PU	Combination of regularised regression, robust-to-outlier, boosting, Random Forest and Gaussian Process	RMSE	4.5	Multiple Imputation	10-fold cross validation	

*PHQ* Patient Health Questionnaire, *QIDS* Quick Inventory of Depressive Symptomatology, *nr* not reported, *S* sleep, *PA* physical activity, *CR* circadian rhythm, *So* sociability, *L* location, *PU* phone use, *SC* skin conductance, *ST* skin temperature, *HR* heart rate, *Li* light, *Cl* clinical data, *SVM RBF* support vector machine-radial basis function, *NRMSD* normalised root-mean-square deviation, *RMSE* root-mean-square error, *MAE* mean absolute error, *STL* single-task learning, *MTL* multi-task learning.

## Discussion

We sought to summarise the literature on passive sensing for depression, in order to map the methodological challenges and threats to reproducibility, in an effort to generate standards in the literature that allow for quantitative synthesis of results. We also assessed the available evidence for a relationship between sensor data and mood to identify leading digital signals for depression.

The first methodological shortcoming stems from the recency of this field. Studies have mostly employed opportunistic study designs, with small sample sizes, short follow-up windows and many being conducted on students, which limits generalisability. Different features may reach peak predictability of mood with different sampling timeframes, so shorter follow-ups may harm the prediction abilities for some behaviours^22^. This is presumably more likely in feature types such as sleep and circadian rhythm which benefit from having more aggregated baseline data^49^. There is no consensus on the timeframe window for optimal phenotyping, different windows, therefore, need to be evaluated.

A critical source of heterogeneity comes from the multitude of methods to create any individual feature, often without providing reasonable details of the process. A feature of sleep quality, for instance, defined in different studies as “Nocturnal Awakenings”, may have been constructed by measuring counts of awakenings, total number of minutes awake, or a proportion of awake vs. asleep in a sleep session. Additionally, there may be differences in how raw sensor data is used to classify an event as sleep or awake. This heterogeneity challenges the ability of investigators to reproduce findings and hampered our ability to summarise results in a meta-analysis.

The exploratory nature of many of these studies means that many different versions of the same feature may have been generated but studies do not transparently describe and justify feature selection and its association with depression. Researchers should provide a description of the feature, in the paper or supplement materials, that is sufficiently clear to allow for appropriate reproducibility.

Additionally, due to the large number of variables obtained in sensing studies, it is likely that published papers are selective in their reporting, and typically emphasise “positive” findings over “negative” ones. Preregistering studies and analyses would be one way of handling this. As the field matures and more studies are published, issues of rigour and reproducibility become more salient, and preregistration becomes more important to reduce reporting bias and cherry-picking in the field.

The sources of heterogeneity arise from varying data collection timespans, depression assessment measures, feature construction, and analytical methods. Whilst differences in these areas represent a healthy heterogeneity in an evolving field, it means that nuance is required in interpreting the presence or absence of a relationship between any specific signal and depressed mood. For example, many studies recruited students, who have different socialisation patterns and smartphone usage to older adults^12,50^. Prediction models based on younger populations have been found not to transfer to older age groups^51^. Further, a signal detected in a clinical sample consisting of people with relatively severe depression may not be reproduced in a population sample where the majority of the sample have few or no depressive symptoms and there may be less variability in key sensor data (e.g. sleep or activity data).

For any broad concept (e.g. sleep or circadian rhythm) different sensor types or operating systems were used, and component features were derived using different approaches. For example, both iPhone and Android smartphone operating systems were included, and sometimes showed differences in significance levels for the same variables^5,18^. This could be due to differences in sampling and data collections for both operating systems, or differences in the user profiles of these products^52^.

We found significant shortcomings in the literature in terms of fundamentals of reporting, including the most basic descriptors of sample characteristics, recruitment, attrition, and missing data. Whilst many of these shortcomings would be resolved by authors and journals following established reporting conventions (e.g. STROBE guidelines), there are a number of issues that are specific to this field.

One of those issues is missing data. Our quality assessments reflect poor reporting of missing data at both the sample level (e.g. attrition and study non-completion) and individual level (e.g. missing sensor data from participants). Missing data can arise from issues with technology, such as device and system failures, or from user-related issues which may be associated with depressed mood. For missing data to be used informatively, these two types need to be identified and dealt with in different ways in terms of their exclusion or analysis. Additionally, researchers set different thresholds as to what counts as missing data. This varies between studies and generates an important threat to reproducibility, making it crucial that these thresholds are reported. Our recommendation is that papers should clearly state how much data were missing and how it was managed in the analysis.

Remote sensing is a relatively new technology that potentially places a considerable burden on study participants—it was therefore surprising that few studies reported on the acceptability of the study protocol to participants. Where this did happen the emphasis was more on evaluating active questionnaire data rather than passive data and device use, where arguably greater issues over privacy and acceptability arise^6,41^.

Finally, there is a general lack of discussion around the extent to which the devices used in these research studies are valid or reliable tools to detect the behaviours of interest. While some behaviours may appear relatively simple to infer from single sensors, such as GPS sensors to infer location and accelerometry as a measure of movement and physical activity, there are validity and reliability concerns surrounding them. For example, although GPS receivers are generally good at detecting location and movement^53^, smartphone-based GPS receivers may differ in their measures of distance travelled^54^. Accelerometers are also generally accepted as reliable but can vary in their output and validity in measuring physical activity across devices^55^.

More complex behaviours such as sociability and sleep require multisensory data and a larger inferential leap. The evidence for actigraphy for the detection of sleep is uncertain, as several studies have found strong correlations between actigraphy and the gold standard of polysomnography (PSG)^56,57^, but a scoping review of 43 studies finding only moderate to poor agreement^58^. A more recent systematic review, however, found that while actigraphy tended to overestimate sleep and underestimate wake, this inaccuracy was consistent, thereby maintaining its usefulness as a potential marker of sleep–wake patterns^59^.

There is a clear gap in the definition of validity and reliability of these devices, however, whether or not these sensors measure the exact ground truth may be less concerning than whether the features we do extract are consistent against each other and serve the purpose of detecting changes in health status. So even though we would expect less reliable technologies to increase the noise to signal ratio, the extent to which any inaccuracies in the devices reduce the strength of association in depression is unknown.

### Association between mood and digital features

Given the heterogeneity in research quality and reporting standards across studies, making inferences from aggregated associations between digital features and mood may be misleading. It would, however, be a missed opportunity to ignore growing consensus between studies in detecting associations between mood and digital features. We, therefore, report a synthesis of the findings but urge the reader to interpret this summary with caution.

Features that consistently appear to be associated with depression are location-based features, with homestay and entropy both associated with the mood in 4 and 5 studies, respectively. However, these studies do not determine the direction of causality, i.e. whether changes in sensed features such as homestay are merely a reflection of behaviours that appear in depression, such as reduced physical activity and social withdrawal^60,61^ or whether they are, in themselves, predictors of deterioration in mood.

Several sleep features appear also to be consistently associated with depressed mood, with sleep stability showing the highest proportion of significant associations. When measuring socialisation, proximity-related features using Bluetooth and microphone sensors seem more sensitive to mood than call and message frequency counts. However, many of these studies have small sample sizes (median = 58), student samples with a low mean age^34^ or report a high degree of intra- and interindividual variance in daily phone usage^62^. Recent studies with larger and more diverse samples using classification machine learning techniques have found that a low average number and duration of calls made daily predicted depression state^63^.

Even though disruptions in circadian rhythms have been thought to affect depression^64^, the majority of studied features did not have a significant association with mood. As previously mentioned, this may be due to short follow-up since median follow-up times for circadian features = 9 days.

The findings of this review highlight the array of potential predictors that sensor data generates. As such, machine learning methods have been the choice analytic approach to the digital phenotyping of depression from multiple features. In addition to helping account for important interactions between the objective features, for example how the effect of being alone is mediated by location (being indoors vs outdoors)^45^, analysing multimodal data in this way may help cover missing data from one source to another. However, machine learning methods have been criticised for lacking transparency in how the model is built and how individual variables contribute to the overall prediction^65^. Some studies in the current review do report their top predictors and bivariate associations with depression, but the question of how well these models can be replicated remains, highlighting the importance of thorough reporting.

### Strengths and limitations

Our attempt to summarise the literature is necessarily crude because the reporting of feature–depression associations was too opaque and diverse to allow any credible attempt at meta-analysis. We have therefore had to rely on simple counts of associations reported, and this comes with caveats that reports are not weighted by sample size, follow-up duration or study quality. It is possible that the associations we have reported are due to reporting bias, as mentioned in the previous section, where investigators emphasise “significant” findings over “non-significant” ones.

To present low-level features in a clear and meaningful way in this review, we combined them into broader low-level features and therefore some of the nuances between them were lost. For example, if one study extracted two features such as a total number of minutes spent in phone calls and the average length of a phone call, they would both load into Call Duration, within the “Sociability” Feature Type (Supplementary Tables 3–10).

Several studies included in this review have overlapping samples as they come from existing datasets. For example, four papers^26,42,45,48^ use the StudentLife open dataset, where there is some similarity in the analysis, meaning that some of the feature associations may be duplicated.

### Recommendations and conclusions

Whilst there have been attempts at standardising reporting standards for actively collected questionnaire data on mood^66^, and guidelines exist for the reporting of observational data (STROBE^67^) and multivariable prediction models (TRIPOD statement^68^), there is a need to develop consensus over the manner in which such mobile health studies are conducted and reported. This should not come at the expense of stifling innovation and should acknowledge that a new field of study takes time to develop.

The literature we identified derives from both clinical and computer science disciplines and some of the heterogeneity we report results from these disciplines having distinct conventions, with medical outputs putting more weight on sample and clinical outcome characteristics but often overlooking feature extraction and analysis description. The importance of recruiting and reporting the diversity of study samples, however, is highlighted by the difference in validity of these devices in detecting the behaviours of interest. For example, some wearable devices may be more accurate on lighter skin tones^69^, and on men^70^.

There is a need for experts across the disciplines to build upon and generate a consensus on a set of established guidelines, but based on this work, the following recommendations emerge as a first step at attempting to improve the generalisability of research and generate a more standardised approach to passive sensing in depression.

Sample recommendations:

- Report recruitment strategies, sampling frames and participation rates.
- Increase the diversity of study populations by recruiting participants of different ages and ethnicities.
- Report basic demographic and clinical data such as age, gender, ethnicity and comorbidities.
- Measure and report participant engagement and acceptability in the form of attrition rates, missing data, and/or qualitative data.

Data collection and analysis:

- Use established and validated scales for depression assessment.
- Present the available evidence, if any, on the validity and reliability of the sensor or device used.
- Register study protocol including pre-specification of analytical plans and hypotheses.
- Describe in sufficient detail to allow replication, data processing and feature construction.
- Provide a definition and description of missing data management.
- In machine learning models, describe the model selection strategy, performance metrics and parameter estimates in the model with confidence intervals, or nonparametric equivalents (for a full guideline on reporting machine learning models see Luo^71^).

Data sharing considerations:

- Make the code used for feature extraction available within an open science framework.
- Share anonymised datasets on data repositories.

The above points cover aspects of transparency, validity and generalisability. Data sharing considerations become critical in this respect, especially with the use of big data and machine learning models, where validation of the model and data is an integral part of the process. It is therefore important to work towards the creation of open datasets or the widespread sharing of data and to work with community groups to standardise the description, exchange and use of mobile health data.

Our most pressing recommendation, however, is that there is a need for consistency in reporting in this field. The failure to report basic demographic information found in many studies, particularly from the computer science field, and the limited description in feature extraction and analysis in medical papers, have important implications for the interpretation of findings. A common framework, with standardised assessment and analytical tools, robust feature extraction and missing data descriptions, tested in more representative populations would be an important step towards improving the ability of researchers to evaluate the strength of the evidence.

## Methods

### Search strategy and selection criteria

We searched Pubmed, IEEE Xplore, ACM Digital library, Web of Science, and Embase and PsychInfo via OVID, for studies published between January 2007 until November 2019, and used a combination of terms related to the key concepts of (1) depression and (2) digital sensors and remote measurement technologies (RMTs) (full search in the Supplementary Note 1). We also conducted searches based on bibliographies of reviews and meta-analyses on the topic. The protocol was registered on PROSPERO 2019 CRD42019159929.

Studies had to have measured depressive symptoms in either clinical or epidemiological samples and to consist of samples with mean ages between 18 and 65 years, due to the differences in behavioural patterns for older adults and children. We limited studies to those which had extracted data for at least 3 consecutive days (to allow for intraday mood fluctuations) from smartphones and wrist-worn devices. Data from devices not worn on the wrist, e.g. on the chest, upper arm or hip, were excluded due to measurement discrepancies between devices worn in different body parts^72^. Studies had to link data between validated scales of depression severity or status (case/non-case) and digital sensor-based variables including measures of behaviour, e.g. activity, sleep, etc., gathered passively. Studies had to be written in English, German or Spanish because these are the languages spoken by the reviewers, be published, peer-reviewed and with accessible full text.

Studies were excluded if their primary focus related to a condition other than depression as well as those from inpatient settings. Studies focusing specifically on bipolar depression were excluded, however, mixed studies consisting of unipolar and bipolar were included provided unipolar cases comprised a substantial majority (at least 80%) of the sample. We excluded studies published before 2007 as this was when the first smartphones became available.

### Procedure

Studies were checked for eligibility by two researchers independently screening titles and abstracts. Potentially eligible studies’ full texts were reviewed by one researcher, with a second researcher evaluating a random sample of 10% of all texts for validating purposes. Disagreements at any stage of eligibility and data extraction were resolved by discussing with an additional reviewer. Agreement of >90% was reached for all reviewer pairs. The eligibility process was documented according to PRISMA guidelines^73^.

### Data extraction

Data extraction included the following variables: sample characteristics (*N*, mean age, gender, ethnicity), comorbidities, study design, study setting (clinical, community, student), depression outcome measures, length of follow-up, device type, features measured, sensors used, statistical analyses and significance levels.

### Study quality assessment

No single quality assessment tool was suitable because of the ditabversity of study types. We, therefore, combined the Appraisal Tool for Cross-Sectional Studies (AXIS tool^74^ and the Newcastle–Ottawa Scale (NOS) for longitudinal studies^75^. Items were scored with two points for fully fulfilled items, one point for partially fulfilled items, and zero for a non-fulfilled item (see Supplementary Table 11 for a description of each criterion). We added an item regarding having a published protocol prior to publishing results (1 point for a published protocol). Data extraction was carried out on all studies, regardless of their quality assessment score.

### Feasibility

We collected information on five measures of the feasibility of using digital health tools, with the aim of identifying potential obstacles to their implementation: engagement with study devices, reasons for study drop out, reported problems with technology, percentage of study tasks completed, attrition and missing data.

### Data synthesis

Eight categories of behavioural features were identified: sleep, physical activity, circadian rhythm (rest-activity patterns through a 24-h period), sociability, location, physiological parameters, phone use and environmental features. Supplementary Tables 3–10 provide descriptions for each feature. Within each behavioural category, there are lower-level features, which group together several individual features as reported by each study. It was therefore possible for a single study to present multiple associations for the same feature. Significant associations according to 0.05 *p*-value thresholds are presented. Due to the heterogeneity of feature types, study designs and data reporting we did not conduct a meta-analysis.

## Supplementary information

Supplementary Information

## Data Availability

The data that support the findings of this study are available from the corresponding author upon reasonable request.
