# Supplementary material for: Digital health tools for the passive monitoring of depression: a systematic review of methods
Source: NPJ Digit Med. 2022 Jan 11;5:3. doi: 10.1038/s41746-021-00548-8 (PMC8752685; doi:10.1038/s41746-021-00548-8)
Supplement: Supplementary file 1 — Supplementary Information [file 41746_2021_548_MOESM1_ESM.pdf]

SUPPLEMENTARY INFORMATION FOR

Digital Health Tools for the Passive Monitoring of Depression: A Systematic Review of Methods

Valeria De Angel MSc [1,2], Serena Lewis BSc [1,3], Katie White BSc [1], Carolin Oetzmann MSc[1], Daniel Leightley PhD [1], Emanuela Oprea MSc [1], Grace Lavelle PhD [1], Faith Matcham PhD [1], Alice Pace BSc [4], David C Mohr PhD[5,6], Richard Dobson PhD [2,7], Matthew Hotopf PhD [1,2]*

1. Institute of Psychiatry, Psychology and Neuroscience, King's College London, London, UK
2. NIHR Maudsley Biomedical Research Centre, South London and Maudsley NHS Foundation Trust, London, UK
3. Department of Psychology, University of Bath, Bath, UK
4. Chelsea And Westminster Hospital NHS Foundation Trust
5. Center for Behavioral Intervention Technologies, Northwestern University, Feinberg School of Medicine, Chicago, IL, USA
6. Department of Preventive Medicine, Northwestern University, Feinberg School of Medicine, Chicago, IL, USA
7. Department of Biostatistics and Health Informatics, Institute of Psychiatry, Psychology and Neuroscience (IoPPN), King's College London, 16 De Crespigny Park, London, SE5 8AF, UK

* Corresponding Author contact details:

E3.08, 3rd floor East Wing, Institute of Psychiatry, Psychology and Neuroscience

16 De Crespigny Park, London, SE5 8AF

E: valeria.de_angel@kcl.ac.uk

Supplementary Table 1

Supplementary table 1 – the devices and models used to measure behaviour.

Studies	Device	Model(s)	Behaviour	Sensors
28, 32, 36, 38, 39, 43, 44, 45, 46, 77, 84, 90, 91, 92, 97	Actiwatch Mini Mitter Co. Inc.	2, Score, Spectrum, 64	Sleep	Accelerometer, Light
		1, 2, Spectrum, 64	Circadian Rhythm	Accelerometer, Light
		2	Physical Activity	Accelerometer, Light
87,	Actillume® Actigraph		Sleep	Accelerometer, light
			Physical Activity	Accelerometer, Light
34, 83	Actiwatch 4, Cambridge Technology Ltd	4	Sleep	Accelerometer, Light
		4	Circadian Rhythm	Accelerometer, Light
		4	Physical Activity	Accelerometer, Light
29	Actiwatch, Philips Respironics, USA	64, 2, L	Sleep	Accelerometer, Light
		64, 2, L	Circadian Rhythm	Accelerometer, Light
		2, L	Physical Activity	Accelerometer, Light
24, 25, 82, 91	GENEActiv, Activinsights, UK		Sleep	Accelerometer, Light, Temperature
			Circadian Rhythm	Accelerometer, Light
			Physical Activity	Accelerometer, Light
76, 93, 95	Motionlogger, Ambulatory Monitoring, Ardsley, NY.	Basic Mini, Micro Mini, Octagonal Basic.	Sleep	Accelerometer, Light
			Circadian Rhythm	Accelerometer, Light
6	Empatica	E4	Sleep	Accelerometer, Light, Electrodermal activity, Skin temperature, Heart Rate
		E4	Physical Activity	Accelerometer, Light, Electrodermal activity, Skin temperature, Heart Rate
41, 85	ActiGraph Link accelerometer	GT9X, GT3x	Physical Activity	Accelerometer, Light
81	Garmin		Physical Activity	Gyroscope, Accelerometer, Compass
	Tempatilume® (Cebrasil, Inc. Brazil)		Circadian Rhythm	Accelerometer, Light
42			Physical Activity	Accelerometer, Light
			Environmental	Light, temperature
94	Pedometer	StepCount SC-01	Physical Activity	Pedometer
5, 81	Fitbit	Charge HR	Sleep	Accelerometer, Light, Heart Rate
		Charge HR	Location	GPS
		Charge HR, unknown model	Physical Activity	Accelerometer, Light, Heart Rate
96	Microsoft Band	2	Physiology	Heart Rate
5, 6, 7, 22, 31, 40, 47, 49, 50, 51, 52, 81, 89, 96	Smartphone	Android	Sleep	Light sensor, microphone, screen activity, accelerometer
		Android	Circadian Rhythm	light sensor, microphone, screen activity, accelerometer
		Android + iOS	Location	GPS signal
		Android + iOS	Location	GPS + Bluetooth
		Android + iOS	Location	GPS + Wifi Signal
		Android + iOS	Location	Screen unlock duration
		Android + iOS	Location	Screen unlock times
		Android + iOS	Physical Activity	App usage

		Android + iOS	Physical Activity	Accelerometer
		Android + iOS	Physical Activity	GPS
		Android + iOS	Physical Activity	Pedometer
		Android + iOS	Socialisation	Combination: GPS + smartphone Wifi logs
		Android + iOS	Socialisation	Call logs
		Android + iOS	Socialisation	SMS logs
		Android + iOS	Socialisation	GPS
		Android + iOS	Socialisation	Microphone
		Android	Environmental	Bluetooth
		Android	Environmental	Humidity

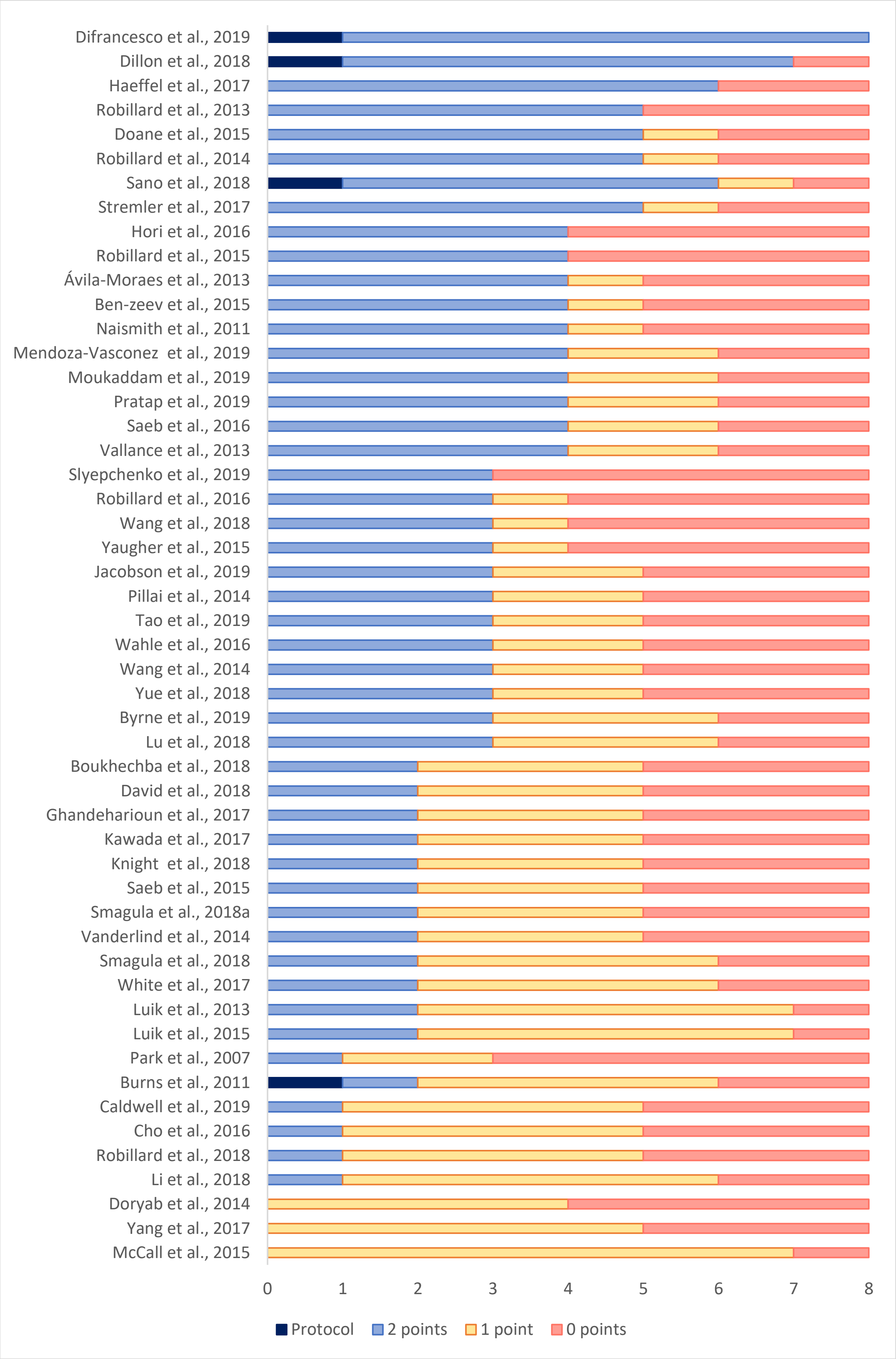

Supplementary Figure 1 | Quality Assessment Score Distribution. Figure showing the distribution of scores on all eight quality assessment items, including whether authors refer to a published protocol, in order of highest to lowest quality. Each row shows how many items were given a score of 2, 1 or 0 points. Presence of a protocol was scored either 1 (if present) or 0 (if absent).

Supplementary Table 2

Supplementary table 2 – raw and percentage quality assessment scores per study. Total score was calculated out of 15.

Field	Reference	Total Score (/15)	% score
Medical	Difrancesco et al., 2019	15	100.0
Medical	Dillon et al., 2018	13	86.7
Medical	Haeffel et al., 2017	12	80.0
Medical	Sano et al., 2018	12	80.0
Medical	Doane et al., 2015	11	73.3
Medical	Robillard et al., 2014	11	73.3
Medical	Stremler et al., 2017	11	73.3
Medical	Mendoza-Vasconez et al., 2019	10	66.7
Medical	Moukaddam et al., 2019	10	66.7
Medical	Pratap et al., 2019	10	66.7
Medical	Robillard et al., 2013	10	66.7
Medical	Saeb et al., 2016	10	66.7
Medical	Vallance et al., 2013	10	66.7
Computer Science	Ávila-Moraes et al., 2013	9	60.0
Medical	Ben-zeev et al., 2015	9	60.0
Computer Science	Byrne et al., 2019	9	60.0
Medical	Lu et al., 2018	9	60.0
Medical	Luik et al., 2013	9	60.0
Medical	Luik et al., 2015	9	60.0
Medical	Naismith et al., 2011	9	60.0
Medical	Hori et al., 2016	8	53.3
Medical	Jacobson et al., 2019	8	53.3
Medical	Pillai et al., 2014	8	53.3
Medical	Robillard et al., 2015	8	53.3
Computer Science	Smagula et al., 2018b	8	53.3
Medical	Tao et al., 2019	8	53.3
Medical	Wahle et al., 2016	8	53.3
Medical	Wang et al., 2014	8	53.3
Medical	White et al., 2017	8	53.3
Medical	Yue et al., 2018	8	53.3
Computer Science	Boukhechba et al., 2018	7	46.7
Medical	Burns et al., 2011	7	46.7
Medical	David et al., 2018	7	46.7
Medical	Ghandeharioun et al., 2017	7	46.7
Medical	Kawada et al., 2017	7	46.7
Medical	Knight et al., 2018	7	46.7
Medical	Li et al., 2018	7	46.7
Medical	McCall et al., 2015	7	46.7
Computer Science	Robillard et al., 2016	7	46.7
Medical	Saeb et al., 2015	7	46.7
Medical	Smagula et al., 2018a	7	46.7
Medical	Vanderlind et al., 2014	7	46.7
Medical	Wang et al., 2018	7	46.7
Medical	Yaugher et al., 2015	7	46.7
Computer Science	Caldwell et al., 2019	6	40.0
Medical	Cho et al., 2016	6	40.0
Medical	Robillard et al., 2018	6	40.0
Medical	Slyepchenko et al., 2019	6	40.0
Medical	Yang et al., 2017	5	33.3
Medical	Doryab et al., 2014	4	26.7
Medical	Park et al., 2007	4	26.7

Supplementary Table 3

Supplementary table 3 – Feature Descriptions for Sleep features extracted in all included studies.

Low-Level Features	Description	Study-level feature examples
**Total Sleep Time**	The amount of actually sleep time in a sleep episode; this time is equal to the total sleep episode less the awake time	Total Sleep Time (TST)<br>Total Sleep Duration Per Night<br>Awake Duration
**Sleep Quality**	A combination of factors which relate to how much of the time that is intended for sleep is actually spent sleeping.	Intradaily Variability In Sleep<br>Wake After Sleep Onset<br>Number Of Nocturnal Awakenings<br>Minutes After Wakeup
**Sleep Efficiency**	Sleep efficiency is another measure of sleep quality presented independently due to its popularity. It is the percentage of time spent asleep while in bed. It is calculated by dividing the amount of time spent asleep (in minutes) by the total amount of time in bed (in minutes).	Sleep Efficiency
**Sleep Stability**	Features of variability in sleep	Sleep Start Time Variability<br>Interdaily Stability In Sleep<br>Sleep Variability Standard Deviation<br>Sleep Onset Variability
**Sleep Architecture**	The basic structure of sleep	Mean Mid-Sleep Time<br>Acrophase Of Sleep<br>Mid Sleep On Free Days<br>Mean Mid-Sleep Time
**Sleep Onset Latency**	The amount of time it takes you to go from being fully awake to sleeping	Sleep Onset Latency (SOL)<br>Sleep Onset Latency (Women)<br>Sleep Onset Latency (Men)
**Sleep Onset**	The time at which sleep onset happens	Sleep Onset
**Sleep Offset**	The time at which the individual awakens.	Sleep Offset
**Time In Bed**	Total amount of time spent in bed	Time In Bed

Supplementary Table 4

Supplementary table 4 – Feature Descriptions for Physical Activity features extracted in all included studies.

Low-Level Features	Description	Study-level feature examples
**Activity Time**	Time spent engaging in physical activity	Summation Of All Active Periods<br>Fraction Of Time In Motion<br>Minutes Per Week Of Physical Activity<br>Average Wrist Activity/Min
**Activity Levels**	General levels of activity	Average 24-Hour Activity<br>Gross Motor Activity Per Day<br>Standard Deviation Motion<br>Average Motion
**Intensity**	Activity features that differentiate between light, moderate and vigorous activity.	Minutes Of Heart Rate In Fat-Burn Zone<br>Time In Moderate-To-Vigorous Physical Activity<br>Time In Light Physical Activity
**Speed**	Movement speed	Speed Mean<br>Speed Variance<br>Average Moving Speed
**Sedentary Time**	Total time spent doing no activity	Sedentary Minutes<br>Time In Sedentary Behaviours<br>Stationary Time (Mean)
**Step Count**	Step count	Step Count

Supplementary Table 5

Supplementary table 5 – Feature Descriptions for Circadian Rhythm features extracted in all included studies.

Low-Level Features	Description	Study-level feature examples
**Hour Based Activity Levels**	Activity levels at different times of day	Motor activity (diurnal)<br>daytime activity levels(DALs) (am)<br>daytime activity levels(DALs) (pm)<br>mean activity during active period (day)
**Intradaily Stability**	The ratio of the hour-to-hour activity variability to the overall activity variability (higher values reflect more fragmented rhythms, e.g., due to frequent daytime napping or night-time awakenings.	Intradaily stability<br>Intradaily variability<br>change in intra-daily variability (IV),
**Acrophase**	peak of activity: a measure of the timing of overall high values recurring in each cycle, expressed in (negative) degrees in relation to a reference time set to 0°, with 360° equated to the period; and the period is the duration of one cycle	Acrophase
**MESOR**	MEAN activity levels: a rhythm-adjusted mean	MESOR
**Amplitude**	difference between peak and troughs of activity: difference between most active time and the least active time of the day, (higher values indicate a greater RAR amplitude) a measure of the extent of predictable change within a cycle	Relative amplitude
**Circadian Rhythmicity**	The coefficient of determination (or R2; not illustrated here), a measure reflecting the goodness of fit,<br>was used as an indicator of circadian rhythmicity.	Circadian Rhythmicity
**Transition Probabilities**	The probability of transitioning from active to rest state or vice versa	Active to Rest - day<br>Active to rest - night<br>Rest to Active - night
**Interdaily Stability**	Ratio of variability within the mean 24-hour activity profile to the overall activity variability (higher values indicate greater stability of the mean 24-hour profile across days)	Interdaily stability
**Alpha**	Relative width of the curve at the middle of the peak. Higher alpha indicates relatively narrower active periods compared to rest periods.	Alpha
**Period**	The time in between activity peaks, usually 24 hours. Shorter periods lead to behaviour occurring at an earlier clock time and long periods to later timing	Period
**Beta**	Indicator of the steepness of the rise and fall of the curve, indicative of a faster transition from rest to active.	Beta

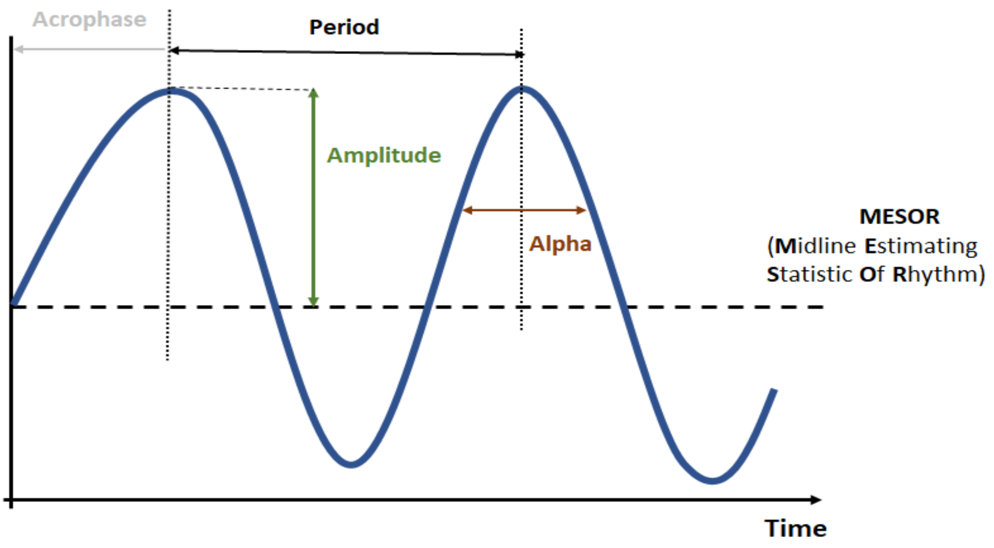

Supplementary Figure 2 | Circadian Rhythm terms. A graph showing a cosine wave representing the circadian curve with the period of 24 h and its terminology.

Supplementary Table 6

Supplementary table 6 – Feature descriptions for Sociability features extracted in all included studies.

Low-Level Features	Description	Study-level feature examples
Frequency of Phone Interactions	Frequency of Phone Calls of Text Messages	Daily Call count<br>SMS count<br>Average frequency of calls per day
Call Duration	Call duration	Call duration
Missed Interactions	Unreturned calls	Unreturned calls<br>Missed interactions
Speech Duration	Length of detected speech	Speech duration<br>Conversation duration during day<br>Change in Conversation duration (slope)
Socialisation By Proximity	Detected proximity to others by nearby Bluetooth devices of speech.	Location/noise/voice<br>Socialisation by proximity and noise
Conversation Frequency	Number of times conversation was detected nearby	Conversation frequency during day<br>Conversation frequency during evening
SMS Length	SMS length	SMS length
Unique Remote Interactions	Total number of unique individuals with whom a participant interacted through phone calls or SMS messages on a particular day	Interaction diversity
Time Spent On Messages	Total time spent using messages	Total time spent using messages

Supplementary Table 7

Supplementary table 7 – Feature descriptions for Location features extracted in all included studies.

Low-Level Features	Description	Study-level feature examples
Entropy	The variability of time the participants spend at a certain location. High entropy translates to spending time more uniformly across different locations.	Entropy<br>Normalised Entropy
N of Locations	The number of locations visited	Number of unique locations<br>Total standard deviation of location<br>Number of clusters

Home Stay	Amount of time spent at the location identified as Home	Homestay
Location Variance	The variability in a participant's location	Location Variance
Average Moving Speed	Average Moving Speed	Average Moving Speed
Time At Location	Average amount of time spent in a particular location.	Average staying time per visit across the study<br>Cumulative staying time across the study<br>Time at on-campus health facilities(mean)
Total Distance	Total Distance Travelled by a participant	Total Distance
Transition Time	The percentage of time during which a participant was in a non-stationary state. This was calculated by dividing the number of GPS location samples in transition states by the total number of samples.	Transition Time
Mobility Radius	The radius of the area within which a person moved.	Mobility Radius

Supplementary Table 8

Supplementary table 8 – Feature descriptions for Phone Use features extracted in all included studies.

Low-Level Features	Description	Study-level feature examples
Unlock Duration	The amount of time a person's phone is unlocked and therefore in use. Commonly referred as screen-time.	Total phone usage duration<br>Mean phone usage duration at student accommodation
Specific App Use	The types of apps used.	total time spent using Instagram<br>total time spent using maps<br>total time spent using photo app
Unlock Frequency	The number of times a phone is unlocked.	phone usage frequency
Combination Of Phone Use	Combination of smartphone use features	combination of all smartphone use features

Supplementary Table 9

Supplementary table 9 – Feature descriptions for Physiology features extracted in all included studies.

Low-Level Features	Description	Study-level feature examples
Temperature	Temperature recorded from skin.	Diurnal Peripheral temperature<br>Amplitude of temperature rhythm<br>Mean elevated temperature time
Heart Rate	The number of heart beats per minute	Heart rate
Electrodermal Activity	Skin conductance	Electrodermal activity<br>Difference in number of skin conductance level peaks

Supplementary Table 10

Supplementary table 10 – Feature descriptions for Environmental features extracted in all included studies.

Low-Level Features	Description	Study-level feature examples
Humidity	Environmental humidity	Humidity - males<br>Humidity - females
Light	Ambient Light	Amplitude of light intensity<br>Acrophase of light<br>Mean elevated light time

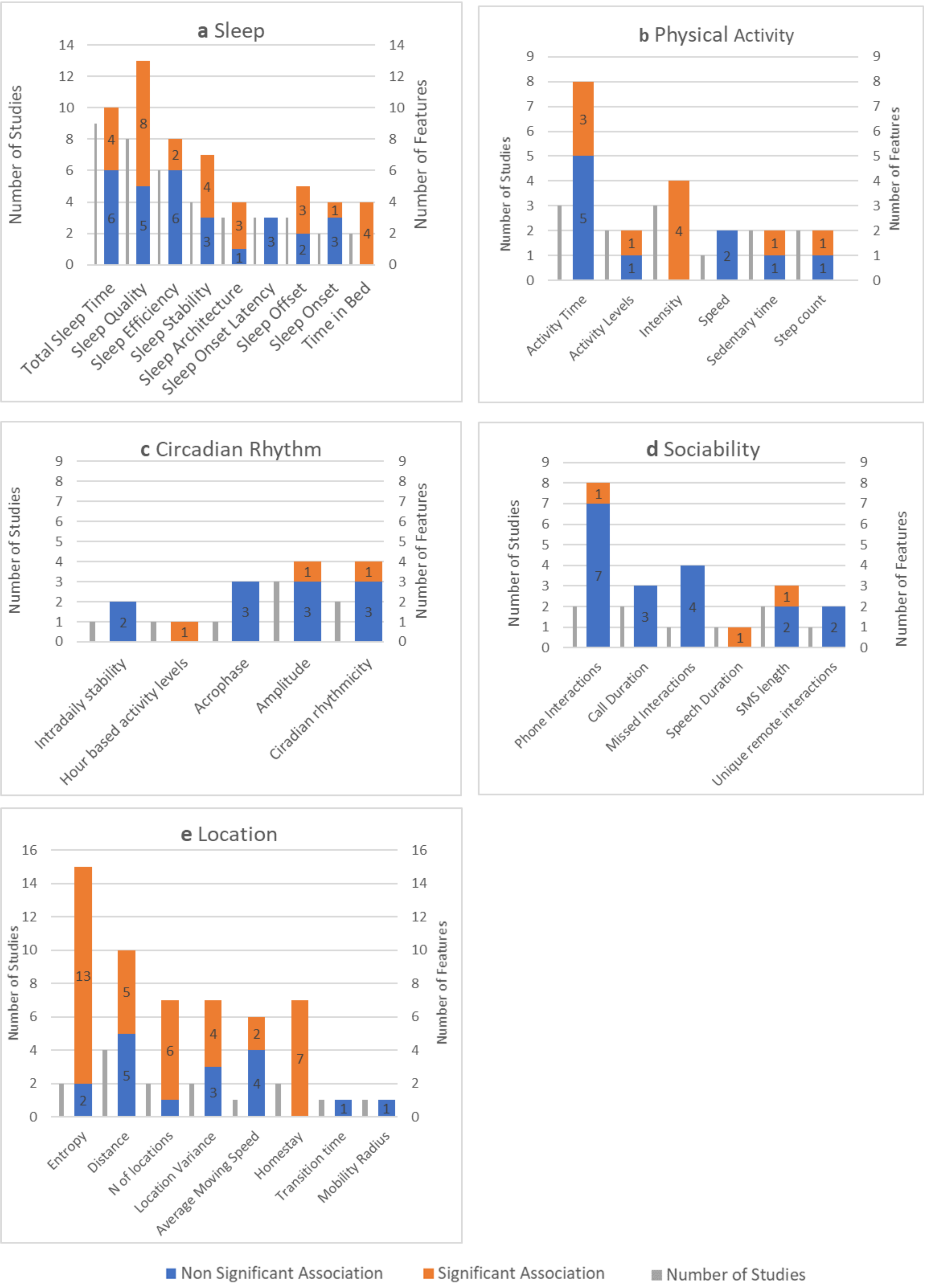

Supplementary Figure 3 | Sensitivity analysis. Data synthesis for included studies scoring higher than 8 on the quality assessment scale. The number of times each feature (**a** sleep, **b** physical activity, **c** circadian rhythm, **d** sociability, and **e** location) has been reported in all included studies and their association with depression, where these associations are defined as having a below-threshold p-value ("Significant Association"), above-threshold p-value ("Non-Significant Association"), and where statistical methods have been used that do not yield p-values ("Non p-value"). Only one study looking at Phone Use scored over 8, a multiparametric study with no bivariate associations, and was therefore not included above. The graphs also show the number of studies assessing each feature.

Supplementary Note 1: Full search strategy

The following databases were searched: 1) Pubmed, 2) Embase, PsychInfo via OVID, 3) IEEE Xplore, 4) ACM Digital library, 5) Web of science,

We included terms surrounding the following key concepts:

1. Depression, depressive disorder
2. RMTs, sensors, technologies (Portable or wearable technology)

	Keywords	Ti/Ab
1	mood disorder; affective disorder; depression; depressive mood symptoms, mental health, depress*, Unipolar affective disorder, mental disorders	remote emotional health monitoring system, mood,
2	"Objective Behavioral Features", objective features; sensor data; "smart phone"; wearable devices; wearable, smartphone, app, apps, accelerometer, pedometer, actigraphy, motor activity, Psychomotor activity, Acceleration, Heart rate, heart rate and movement sensor, "digital biomarker", digital phenotype	activity measurement, wrist-worn, remote, Psychomotor activity, objectively measured activity parameters, Electronic monitoring, objective measure

1) Pubmed search:

	Depressive Disorder[Mesh] OR Major Depressive Disorder[Mesh] OR Depression[Mesh] OR depressi*[Title/Abstract] OR "affective disorder"[Title/Abstract] OR "mood disorder"[Title/Abstract]
AND	Remote Sensing Technology [MeSH] "digital"[Title/Abstract] OR smartphone[Title/Abstract] OR mobile[Title/Abstract] OR wearable[Title/Abstract] OR "objective measure"[Title/Abstract] OR "sensor data" OR "wearable devices" OR "smart phone" OR app OR apps OR "activity measure" OR accelеromet* OR pedomet* OR actigraph* OR "psychomotor activity" OR "remote monitoring" OR "GPS" OR "global positioning system" OR "mobile sensor" OR "RMT" OR "remote measurement technologies" OR mHealth OR "digital biomarker" OR "digital phenotype"

Limit to 2007

2) OVID: PsychInfo and EMBASE

exp Major Depression/

major depression/ or affective disorder/ or depressive disorder.mp.

("depressive mood" or "depressed mood" or "depressive symptoms" or "depressed symptoms" or "affective symptoms" or "mood disorder" or depression).ti,ab.

(digital or smartphone or mobile or wearable or objective measure).ti,ab.

("sensor data" or "wearable device" or "smart phone" or smartphone or accelerometer or pedometer or actigraphy or "psychomotor activity" or "remote monitoring" or "GPS" or "global positioning system" or "mobile sensor" or "RMT" or "remote measurement technologies" or mhealth or "machine learning" or app or apps or "activity measure" or "digital biomarker").mp.

limit to yr="2007 -Current"

1	major depression/ or affective disorder/ or depressive disorder.mp.	262002
2	("depressive mood" or "depressed mood" or "depressive symptoms" or "depressed symptoms" or "affective symptoms" or "mood disorder" or depression).ti,ab.	716408
3	1 or 2	795172
4	(digital or mobile or wearable or objective measure).ti,ab.	318272
5	("sensor data" or "remote sensing technology" or "wearable device" or "smart phone" or smartphone or accelerometer or pedometer or actigraphy or "psychomotor activity" or "remote monitoring" or "GPS" or "global positioning system" or "mobile sensor" or "RMT" or "remote measurement technologies" or mhealth or "machine learning" or app or apps or "activity measure" or "digital biomarker").mp.	187037
6	4 or 5	485984
7	3 and 6	10688
8	limit 7 to yr="2016 -Current"	4635
9	limit 7 to yr="2007 - 2015"	4321
10	remove duplicates from 9	3526
11	remove duplicates from 8	3929
12	10 or 11	7455

3) IEEE Xplore:

("remote sensing technology" OR "psychomotor activity" OR "RMT" OR "mhealth" OR "accelerometer" OR "pedometer" OR "actigraphy" OR "sensor data" OR "sensing technology" OR "GPS" or "global positioning system" OR "mobile sensor" OR "smartphone" OR "mobile" OR "wearable" OR "smart phone" OR "app" OR "apps" OR "digital biomarker*" OR "digital phenotype") AND ("depression" OR "depressed mood" OR "depressive symptoms" OR "affective disorder" OR "mental health" OR "mood disorder" OR "mood")

Limit to 2007 onwards

4) ACM Digital library,

NOTE: adding “depressive mood” and/or “depressed symptoms” does not improve the search

"depressive mood" or "depressed mood" or "depressive symptoms" or "depressed symptoms" or "affective symptoms" or "mood disorder" or depression or "major depression" or "affective disorder" or "depressive disorder"

AND

"remote sensing technology" or "sensor data" or "wearable device" or "smart phone" or "smartphone" accelerometer or pedometer or actigraphy or "psychomotor activity" or "remote monitoring" or "GPS" or "global positioning system" or "mobile sensor" or "RMT" or "remote measurement technologies" or mhealth

"filter": {"publicationYear":{ "gte":2007 }},

{owners.owner=GUIDE}

5) Web of science,

# 9	3,689	#8 *Indexes=SCI-EXPANDED, SSCI, A&HCI, CPCI-S, CPCI-SSH, ESCI Timespan=2007-2019*
# 8	4,305	#7 AND #4 *Indexes=SCI-EXPANDED, SSCI, A&HCI, CPCI-S, CPCI-SSH, ESCI Timespan=1900-2019*
# 7	327,872	#6 OR #5 *Indexes=SCI-EXPANDED, SSCI, A&HCI, CPCI-S, CPCI-SSH, ESCI Timespan=1900-2019*
# 6	147,948	TI=(wearable OR mobile) *Indexes=SCI-EXPANDED, SSCI, A&HCI, CPCI-S, CPCI-SSH, ESCI Timespan=1900-2019*
# 5	193,432	TS=("remote sensing technology" OR "sensor data" OR "wearable device" OR "smart phone" OR smartphone OR accelerometer OR pedometer or actigraphy OR "psychomotor activity" OR "remote monitoring" OR "GPS" OR "global positioning system" OR "mobile sensor*" OR "sensing technologies" OR "RMT" OR "remote measurement technologies" OR mhealth OR "digital biomarker*" OR "digital phenotype*")<br>*Indexes=SCI-EXPANDED, SSCI, A&HCI, CPCI-S, CPCI-SSH, ESCI Timespan=1900-2019*
# 4	542,297	#3 OR #2 OR #1<br>*Indexes=SCI-EXPANDED, SSCI, A&HCI, CPCI-S, CPCI-SSH, ESCI Timespan=1900-2019*
# 3	16,431	TS=("affective disorder" OR "mood disorder")<br>*Indexes=SCI-EXPANDED, SSCI, A&HCI, CPCI-S, CPCI-SSH, ESCI Timespan=1900-2019*
# 2	224,301	TI=(depress* OR "affective disorder" OR mood)<br>*Indexes=SCI-EXPANDED, SSCI, A&HCI, CPCI-S, CPCI-SSH, ESCI Timespan=1900-2019*
# 1	492,570	TS=(Depressive Disorder OR Major Depressive Disorder OR Depression)<br>*Indexes=SCI-EXPANDED, SSCI, A&HCI, CPCI-S, CPCI-SSH, ESCI Timespan=1900-2019*

Supplementary Table 11

Supplementary table 11 – Study quality assessment criteria, item names and scoring criteria.

	ITEM CRITERIA	ITEM DESCRIPTION AND SCORING
1	**Protocol Published? [Y, N]**	Is a published protocol mentioned? 1= Yes, 0 = No
2	**Definition of Outcomes (Clinical outcomes and Objective Features)**	2. clear and appropriate definition of outcomes (depression and objective measures). 1. unclear or incomplete 0. none reported
3	**Evidence of Selective reporting (data measured but not reported)**	2. analysed data matches study objectives and post hoc analyses clearly defined as such 1. some variables measured not mentioned/ reported in results. 0. Significant results not defined at the outset nor in line with study objectives.
4	**Sample Description and Eligibility Definition**	2. Gives well-defined eligibility criteria, and the sources and methods of selection of participants. 1. Eligibility criteria incomplete or unclear, or clinical-based inclusion of depression defined by self report (and not assessed by clinician). 0. no mention of sampling strategy / eligibility
5	**Statistical Control for Confounding and/or Multiple Comparisons**	2. clear and appropriate . 1. unclear or incomplete 0. none reported
6	**Missing data (Report and management)**	2. clear and appropriate . 1. unclear or incomplete 0. none reported
7	**Representativeness**	2. sample representative of population of interest, 1. potential selection/sampling bias, 0. sample largely different to the populations it aims to study.
8	**Justification of sample size**	2. clear and appropriate justification of sample size. 1. unclear or incomplete explanation for sample size. 0. none reported
